# Fluorination Anodization of *n*‑Type Silicon in the Dark via Schottky-Driven Electron Extraction

**DOI:** 10.1021/acsmaterialsau.6c00002

**Published:** 2026-03-27

**Authors:** Benjamin Tien-Hsi Lee, Chao-Chia Cheng, Chun-Huang Wu, Lin Cheng, Yu-Sheng Chiou, Shu-Cheng Li, Wei-Chi Huang, Chao-Yuan Cheng, Yang-Chun Chiu, Chao-Ching Chiang

**Affiliations:** † Department of Mechanical Engineering, 34911 National Central University, Taoyuan City 320317, Taiwan, Republic of China; ‡ Department of Physics, National Central University, Taoyuan City 320317, Taiwan, Republic of China; § Department of Materials and Mineral Resources Engineering, 34877 National Taipei University of Technology, Taipei 10608, Taiwan, Republic of China; ∥ Department of Mechanical Engineering, 34884 Asia Eastern University of Science and Technology, New Taipei 220303, Taiwan, Republic of China

**Keywords:** photoluminescence, Schottky junction, fluoridation anodization, self-doping, quasi-*p*-type region, quantum confinement

## Abstract

The band structure of semiconductors governs electron exchange, rendering interfacial electron transfer both controllable and intrinsically time-integrated. Because electron transfer affects the formation and rupture of interatomic bonds, it constitutes the main driving force of chemical and electrochemical reactions. Fluorination anodization of *n-*type silicon in the dark is traditionally considered impossible because of the scarcity of the required species—holes. Here, we report that sufficiently sustained bias induces fluorination anodization of *n-*type silicon in complete darkness under standard electrochemical conditions. We attribute this phenomenon to the formation of a Schottky junction at the metal–semiconductor contact. Continuous long-term electron extraction through this interface guides a dynamic charge reconstruction, resulting in hole accumulation and the creation of a provisional inversion layer. Moreover, to validate this Schottky-driven mechanism, we designed a localized laser beam with an irradiation diameter of 1 mm using three distinct wavelengths—corresponding to energies greater than, near, and less than the silicon bandgap—as a probed region during dark electrochemical processing. These experiments reveal that the anodization reaction pathway is controlled by internal carrier redistribution, i.e., self-doped holes. We introduce an “electric-field budget” framework to quantify the cumulative effect of electron transfer and bias duration on electrochemical kinetics. We report that electric-field-induced electron transfer in semiconductors—particularly its time-integrated and cumulative potential dictated by the interfacial energy barrier—enables chemical or electrochemical reactions even without appropriate external excitation.

## Graphical abstract

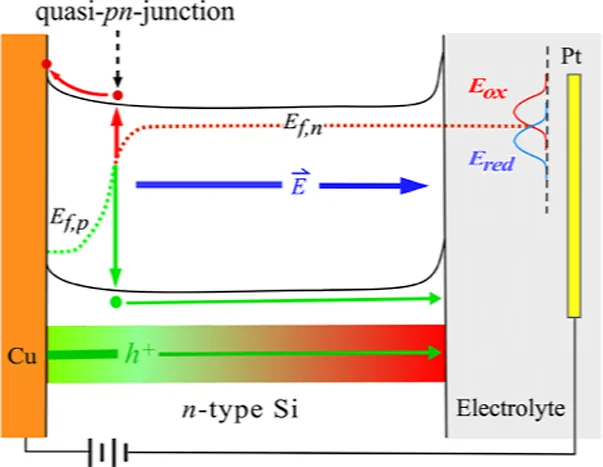

## Introduction

The electrochemical fluorination of silicon has long been a foundational process in semiconductor nanostructuring.^1^ However, a thirty-year synthetic barrier has persisted: the fluorination anodization of *n*-type silicon in the dark is traditionally considered impossible because of the scarcity of minority carrier holes. Current synthetic protocols rely heavily on external illumination to generate the necessary carriers, which results in spatial inconsistencies and limits the scalability of the process.

In this study, we report a fundamentally new synthetic pathway: Schottky-driven dark anodization. Based on the interfacial physics of a Cu/Si Schottky junction, we report that sustained electron extraction guides a dynamic charge reconstruction, resulting in a “hole-self-doping” effect. This process establishes a quasi-p-type inversion layer that sustains the fluorination reaction in complete darkness. To meet the requirement for photoexcitation, we use a unified electrostatic framework that addresses *n*-type semiconductor nanostructure synthesis.

In hydrofluoric acid (HF) electrolytes, fluoride anions do not react directly with silicon because the semiconductor band structure controls interfacial electron transfer. However, an applied bias can significantly modulate the interfacial band structure (band bending) and thus the effective charge-transfer barrier for anodization. However, due to their high electronegativity, fluoride anions tend to physisorb onto silicon surfaces. Once a bias is applied and holes are injected into the silicon, the physisorbed fluoride species are anodized, forming soluble silicon tetrafluoride (SiF_4_). This compound subsequently dissolves into the electrolyte as hexafluorosilicic acid (H_2_SiF_6_), leading to the formation of distinct nanostructures in the anodized regions.^2^ In the 1990s, Canham,^3^ Wilson,^4^ Gösele^5^ and others^2,6,7^ revealed that porous silicon formed via fluorination anodization exhibits photoluminescence (PL), likely due to quantum confinement effects,^8,9^ offering a pathway to synthesize nanocrystals with direct-bandgap-like photonic properties.^2,10–12^ Silicon substrates are inherently indirect-bandgap materials and thus unsuitable for photonic applications, unless internal nanostructures are introduced to modify their band structure. Because of the quantum confinement of the porous silicon structure,^13^ PL generated in anodized regions can be readily visualized under ultraviolet (UV) illumination, providing a straightforward and effective approach for evaluating the optical behavior of the resulting nanostructures for photonic device fabrication.

Here, we define fluorination anodization as an electrochemical process in which fluoride species—including HF molecules and fluorine radicals—participate in the anodic etching of semiconductor surfaces under an applied bias. In this type of process, electric holes (*h* ^*+*^) play a critical role^6,14^ in enabling the anodization reaction in fluoride-based electrolytes. Owing to the significantly greater mobility of electrons—approximately three times greater than that of holes—*n*-type silicon nanocrystals remain highly attractive for silicon photonics applications.

It is widely understood that illumination^6,15^ is essential for anodizing *n-t*ype silicon in hydrofluoric electrolytes; in its absence, electrochemical etching is considered impossible.^6,14,15^ However, when illumination is employed to promote the reaction, the resulting etching profile is highly sensitive to external factors such as light intensity and irradiation distance.^16,17^ Doan et al. used controlled illumination during photoelectrochemical etching to pattern an *n*-type Si(100) surface, producing a porous-silicon layer that exhibits visible photoluminescence. The apparent “color” of the resulting image stems primarily from thin-film optical interference, whereas spatial differences in emission are correlated with illumination-induced local changes in photocurrent and etch rate, enabling the fabrication of a photoluminescent color image on the silicon surface.^6^ Several researchers have attempted to provide holes through alternative means, such as applying external magnetic^16^ or lateral electric fields,^18^ to enable anodization without illumination. Other strategies involve the design of energy-barrier junctions, such as epitaxial *pn* junction structures^17^ or *n–n* ^*+*^ *–n* architectures,^19^ or the incorporation of oxidation–etching pathways using strong oxidizing additives,^20^ to inject holes and facilitate anodization without illumination. One such method, which is based on avalanche breakdown, allows for dark anodization after a sufficiently high voltage is applied.^15,20,21^ However, this approach typically results in the formation of macroporous structures without a PL effect.

In contrast, we propose that anodization can be enabled solely by hole self-doping^22^ induced through the formation of a Schottky junction from the interfacial contact of metal electrode/silicon without illumination. Carraro et al. state that “silicon immersed in an electrolyte behaves as a Schottky diode, a metal–semiconductor contact with the formation of *a* space charge region if it is biased or because of to surface states.”^23^ Based on the fused interfacial characteristics provided by direct wafer bonding processing, a Schottky junction could be established if intimate contact is achieved, enabling the opposing dangling bonds to interact and combine via van der Waals forces.^24^ This hole self-doping mechanism is sustained by Schottky-driven electron extraction, creating a quasi-p-type region near the interface that enables anodization to proceed, even in complete darkness.

Porous silicon formation in HF-based electrolytes follows the well-established Lehmann–Gösele model, encompassing characteristic steps and mechanisms,^2,10,12,14^ which can be summarized by the following chemical reactions (1–3)

1 $${Si}_{\left(s\right)}+{2HF}_{\left(aq\right)}+\left(2-\alpha \right)h^{+}\to {SiF}_{2\left(s\right)}+{2H}_{\left(aq\right)}^{+}+\alpha e^{-}$$

2 $${SiF}_{2\left(s\right)}+{2HF}_{\left(aq\right)}\to {SiF}_{4\left(g\right)}+H_{2\left(g\right)}$$

3 $${SiF}_{4\left(g\right)}+{2HF}_{\left(aq\right)}\to H_{2}{SiF}_{6\left(aq\right)}$$

We propose that, under sustained anodization, electron extraction progressively generates an inversion layer at the silicon–electrolyte interface, which transforms into an electron-deficient, i.e., hole-enriched, quasi-*p*-type region. This reduction in electrons produces an unusual hole self-doping phenomenon, promoting internal hole accumulation. Moreover, the formation of a quasi-*pn* junction between the bulk *n-*type Si and the quasi-*p*-type region promotes hole injection toward the Si/electrolyte interface. The resulting effect enables fluorination-based anodization and nanostructure formation without illumination, as shown conceptually in Figure 1.

**Figure 1:**
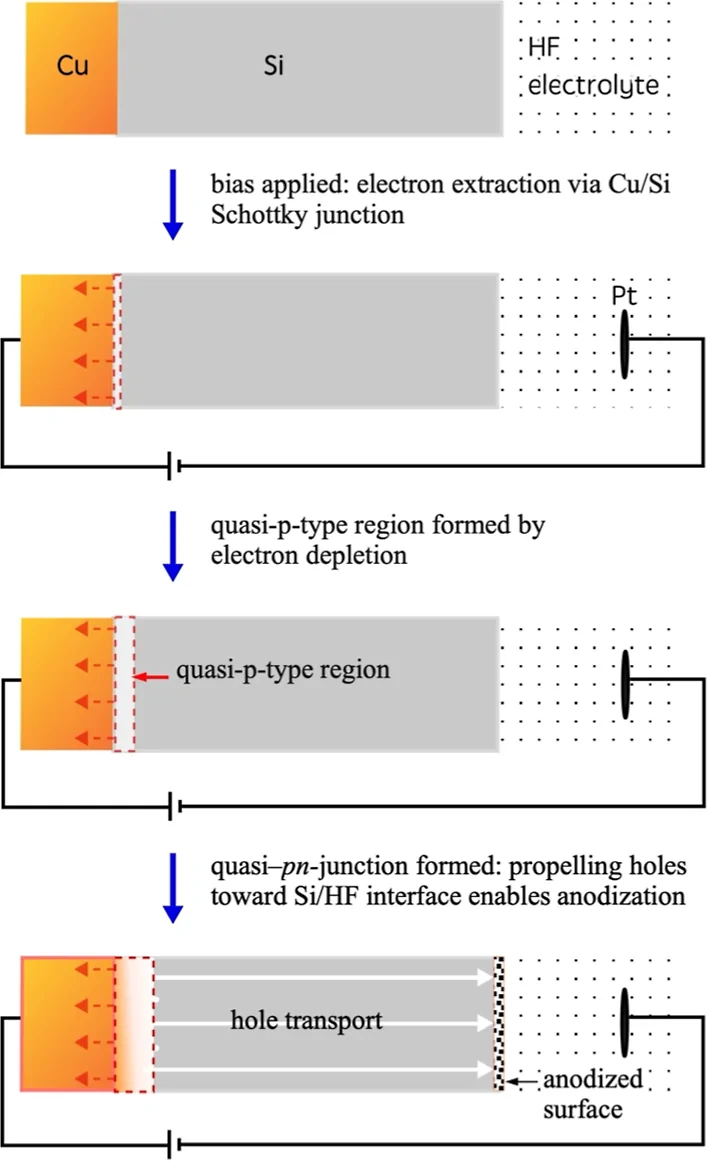
Schematic illustration of the proposed Schottky junction-driven hole self-doping mechanism enabling the fluorination anodization of*n*-type silicon in the dark. (i) Under sustained forward bias, electrons are extracted from the conduction band of *n*-type Si through the Cu/Si Schottky junction. (ii) Electron depletion near the interface induces a quasi-*p*-type region. (iii) The resulting quasi-*pn* junction promotes hole transport toward the Si/HF electrolyte interface, where fluorination anodization proceeds in the absence of illumination.

## Experimental Section

To compare the anodization of *n-*type silicon guided by internally self-doped holes via Schottky-induced electron extraction with that guided by externally injected holes under bandgap-absorbing illumination, we performed electrochemical processing under completely dark conditions on *n*-type Si samples. Silicon samples were prepared by dicing 8” (100), phosphor-doped, *n-*type (1–10 Ω-cm) and *n* ^*++*^-type (0.001–0.005 Ω-cm) prime Si wafers (Wafer Work Co., Taiwan). The electrolyte was composed of a 1:1 volume ratio of HF (CMOS grade, 49%) to C_2_H_5_OH (CMOS grade, 98%). The current density was controlled within the range of 10–100 mA/cm^2^ during standard anodization with respect to porous silicon formation,^10,14,25^ and detrimental effects such as avalanche breakdown or electropolishing were avoided.^21^ To explore the electron extraction induced by the Schottky effect, a comparison laser beam with a diameter of 1.0 mm was used at wavelengths of 633, 1064, and 1550 nm. Figure A1 in the Supplying Information presents a schematic of the electrochemical setup designed to verify the Schottky junction through metal anode contact.

To ensure sufficient electron drainage, the current was kept as constant as possible by adjusting the voltage during electrochemical processing, as shown in the I–V curve in Figure A2 of the Supplying Information. An O-ring was positioned between the silicon surface designated for anodization and the tank wall to prevent electrolyte infiltration into the Si/Cu electrode interface. Then, *n-*type Si wafers (exposed area in the electrolyte = 5.8 cm^2^) were subjected to anodization in the dark at different constant current densities of 17, 52, and 86 mA/cm^2^. The processing times for anodization at each current density were 0.5, 1, 2, and 3 h. To promptly detect the presence of nanocrystal formation, we irradiated the anodized surface with a 10 W ultraviolet (UV) lamp at 365 nm and observed the emergence of photoluminescence.^10^

## Results and Discussion

In contrast to previous studies that did not report anodization after tens of minutes of processing, our *n*-type silicon samples exhibited a uniform porous structure with photoluminescence (PL) across the entire surface after 3 h of fluorination anodization under standard conditions in the dark. Even for heavily doped *n* ^*++*^-type silicon, anodization was also successfully performed under the same conditions but did not exhibit detectable PL emission. After anodization for 3 h, the *n* ^*++*^-type silicon surface turned dark brown, with visible fragments detaching from the surface, indicating the formation of a macroporous structure. The absence of observable photoluminescence is primarily attributed to the effect of high doping concentration, which results in a narrow depletion region that facilitates high-field carrier transport.^26^ This leads to an aggressive etching process causing macropore formation rather than the formation of quantum-confined mesoporous structures. Figure 2 summarizes the results of *n-*type Si anodized in the dark characterized by field emission scanning electron microscope (FE-SEM, JSM-7600F, JEOL, Japan), a micro-Raman and photoluminescence spectroscopy system (T64000, Horiba Jobin Yvon) equipped with a Triax 190 spectrometer and a Syncerity CCD detector, and X-ray diffraction (XRD, D2 Phaser, Bruker, Germany). The surface was fully anodized in the dark both with and without laser irradiation and exhibited red PL (peak location: 600–680 nm) upon UV irradiation. Based on the PL emission wavelength, the diameter of the nanocrystals in the anodized layer was estimated to be 3.2–3.4 nm via the following eq 4.^8^

4 $$E_{PL}^{corr}=E_{0}+\frac{3.73}{d^{1.39}}+\frac{0.881}{d}-0.245$$

where *E*_0_ is equal to 1.12 eV (the bandgap of bulk Si), and *d* is the diameter of the nanocrystal.

**Figure 2:**
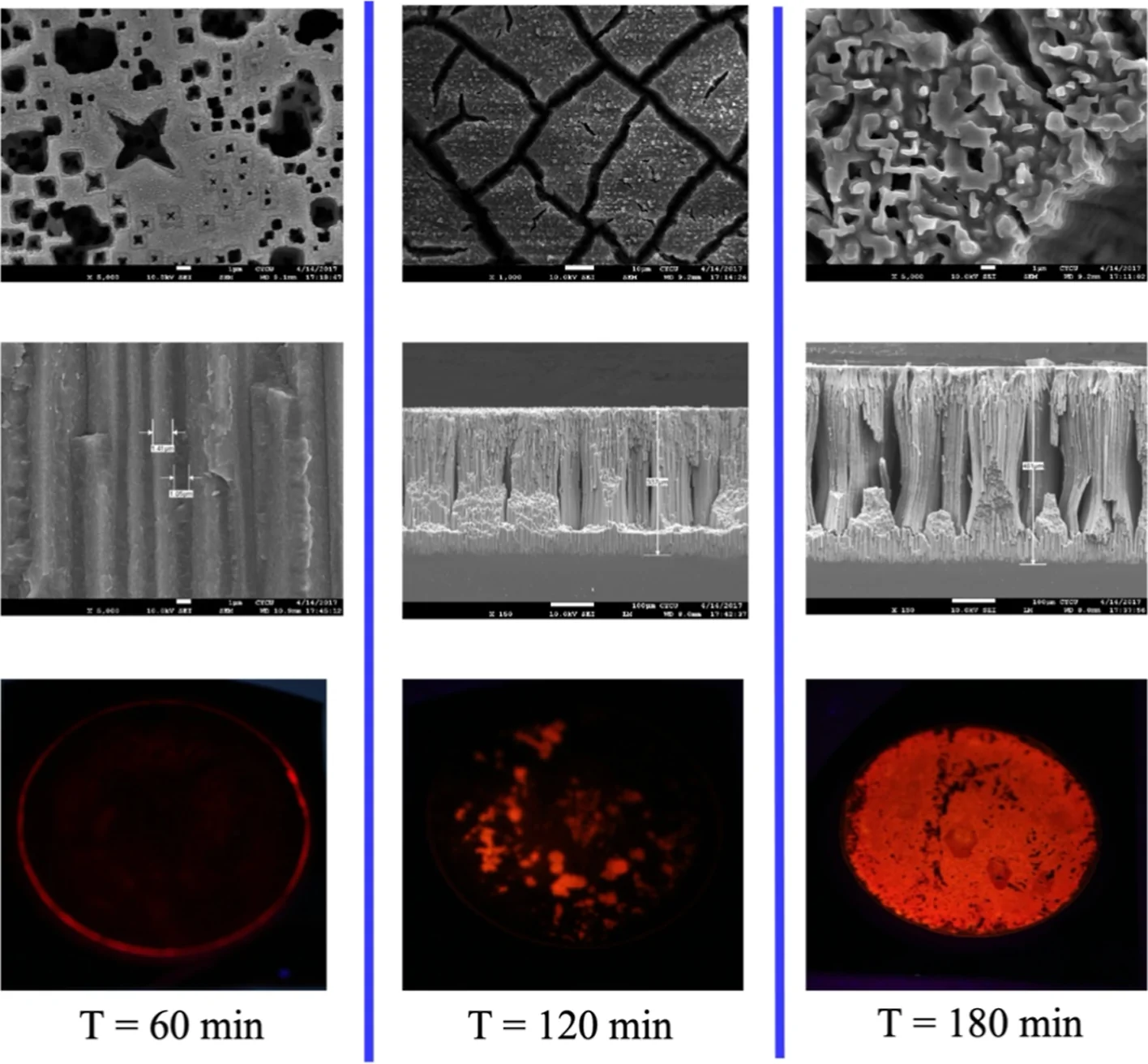
Morphological and photoluminescence (PL) evolution of n-type Si during dark anodization. Surface and cross-sectional FE-SEM images (top/middle) and corresponding PL images (bottom) for samples anodized at 86 mA/cm^2^ for 60, 120, and 180 min.

### Fluorination Anodization of *n*- and *n* ^*++*^-Type silicon in the Dark

As shown in Figure 2, the surface of *n*-type silicon (1–10 Ω·cm) evolves from isolated cross-shaped grooves to vertically aligned porous structures as anodization time increases. Under 250 W UV illumination, red–orange PL initially emerges in localized regions—likely triggered by O-ring pressure—and progressively expands to cover the entire surface. The FE-SEM images of the anodized surface reveal patterns that are nearly identical to those reported in Lehmann’s experiment,^15^ in which the backside of the sample was irradiated with high-intensity light. This suggests that, whether anodized in complete darkness or under illumination, the resulting surfaces consistently exhibit cross-shaped groove structures, a characteristic feature commonly associated with *n*-type silicon. During the first 60 min of anodization, no significant anodization was observed, which is consistent with the expected behavior of *n*-type silicon without external hole injection. In the initial induction period, the substantial anodic current—measured despite the absence of discernible anodization—is primarily consumed by the dynamic charge reconstruction required to establish the quasi-*p*-type inversion layer. Under the influence of the Cu/Si Schottky junction, the applied bias facilitates vigorous electron extraction from the *n*-type silicon conduction band toward the copper electrode. This continuous majority-carrier drainage results in net hole accumulation, effectively transforming the interfacial region into a hole-rich, quasi-*p*-type state. Consequently, the early stage anodic current represents the charge transfer necessary to maintain this inversion-like state and reach the hole density threshold required to initiate the Lehmann–Gösele model etching pathway in complete darkness. Notably, PL was initially observed in a narrow circular region along the tight contact area defined by the O-ring, forming a distinct peripheral ring, as shown in Figure 2 (applied current density = 86 mA/cm^2^). This early stage photoluminescence, which stems from the formation of nanocrystals, may be attributed to localized lattice strain induced by the mechanical pressure of the O-ring, which could facilitate charge accumulation and trigger the onset of anodization. Because the intrinsic carrier concentration varies as exp­(-*E*_*g*_ */2kT*), even a modest reduction in the bandgap (*E*_*g*_) by compressive stress^27^ can significantly increase the local carrier concentration. This bandgap narrowing leads to an increased hole current derived from excess carrier generation within the depletion regions of high-stress areas. During anodization, the initial PL regions appeared as isolated, fragment-like clusters on the surface. As the processing time increased beyond 1 h, these clusters gradually expanded and merged into larger domains. Ultimately, after anodizing for 180 min, the entire surface exhibited uniform PL characteristics, as shown in Figure 2.

After 3 h of anodization at 86 mA/cm^2^, the -type silicon surface exhibits a reddish hue under daylight. This transformation is further validated by the bright red photoluminescence of the scraped powder under UV illumination, with a broad emission peak centered at approximately 680 nm, characteristic of quantum-confined silicon nanocrystals. We used a doctor blade to scrape off the thick anodized layer from the Si and crushed it into powder. The powder exhibited a PL phenomenon when irradiated with UV. Surprisingly, the powder exhibited uniform red–orange photoluminescence under UV irradiation, which remained stable even after the powder was stored at room temperature >3 years. This long-term stability may stem from quantum confinement-induced modifications to the electronic structure,^28^ thereby stabilizing potential surface reconstruction processes.^28,29^ The quantum confinement effect raises the energy levels of dangling surface bonds and reduces their chemical reactivity toward oxygen in the air, thereby increasing the durability of hydrogen termination over extended periods.^30^ While it is possible that hydrogen atoms immediately passivate the surface during anodization to prevent subsequent oxidation,^31^ we also suggest that the fluorine-rich synthesis environment inherent in this process facilitates the formation of a high-quality native oxyfluoride (SiO_*x*_F_*y*_) shell^32^ that encapsulates the nanocrystals. However, the precise evolution of this surface chemistry remains to be definitively mapped through further spectroscopic investigations.

XRD analysis provides direct structural evidence of the porous network formation as shown in Figure 3. A comparison of the XRD patterns of *n*-type Si before and after electrochemical processing confirms that fluoridation anodization proceeded in complete darkness. Specifically, the Si (400) peak associated with the formation of the porous layer exhibits broadening and a slight shift toward a lower angle, appearing at 2θ = 68.8° compared to the bulk Si value of 2θ = 69.1°. This shift indicates the presence of out-of-plane tensile strain induced by the dark anodization process.

**Figure 3:**
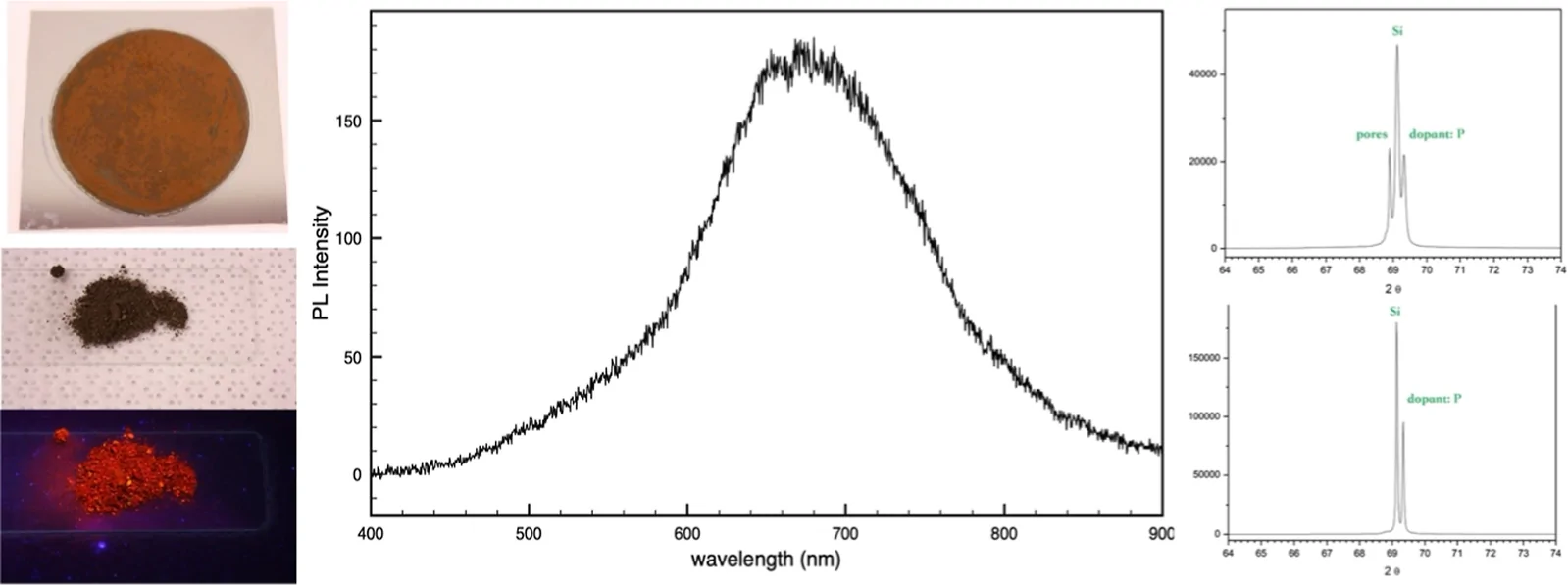
Photoluminescence and structural characterization of porous silicon formed via dark anodization. Daylight and UV photographs of the *n*-type Si (1–10 Ω·cm) surface anodized at 86 mA/cm^2^ for 3 h and its corresponding scraped powder (left), with PL spectrum (center) and XRD patterns (right) confirming nanostructure formation.

Figures A3 and A4 in the Supplying Information show more results for the dark anodization of *n-*type Si observed with the naked eye and FE-SEM.

This result provides direct structural evidence that anodization can proceed under complete darkness—a condition previously considered unsuitable for *n*-type silicon due to the absence of hole carriers.

### Fluorination Anodization of *n*- and *n* ^*++*^-Type silicon in the Dark with Laser Point-Irradiation

We performed anodization of *n*-type Si under laser irradiation at wavelengths of 633, 1064, and 1550 nm. The morphological and optical evolution under localized irradiation was further characterized by specific visual and microscopic transitions. Under a regular fluorescent lamp, the macroscopic surface images captured at 1, 2, and 3 h intervals illustrate the progressive expansion of the anodized front. Remarkably, at the 633 nm laser irradiation spot, the surface developed a distinct black velvet color, signifying the localized destruction of the nanocrystalline material due to excessive etching. Microscopic examination at 200× magnification confirmed this severe structural degradation within the irradiated region after 3 h of processing. Furthermore, spectral analysis provided evidence of the high uniformity of the resulting nanostructures across the substrate. After 2 h of 1550 nm laser irradiation, the PL peak locations recorded at the irradiated spot, the adjacent region, and the remote unirradiated areas were found to be nearly identical, centered at approximately 600 nm. This spectral consistency suggests that while the laser acts as a localized trigger to accelerate the reaction, the fundamental size-tuning of the nanocrystals remains governed by the broader electrochemical environment established through the Schottky-driven mechanism. Representative results are summarized in Figure 4. Under 633 nm laser irradiation for 6 min, only the irradiated point exhibited PL. Under continuous anodization, the PL region gradually extended outward from the irradiated point and eventually distributed to the entire surface after 3 h. Although the area proportion of the irradiated spot to the entire surface was only 0.054%, under a 633 nm laser beam irradiation, the time for the entire surface to emit PL was reduced by 33% of that with anodization in complete darkness. Numerous hot carriers with high kinetic energy accelerate the anodization and were generated by the split of electron–hole pairs because of the bandgap absorption. The laser-irradiated region severely collapsed after 3 h (Figure 4c, 200*X* microscopy observation). The results of 1064 nm laser irradiation are comparable to those of 633 nm laser irradiation, as shown in the Supporting Information. The anodization result with 1550 nm laser irradiation was similar to that with 633 nm laser irradiation, except the irradiating region did not collapse. When a 1550 nm laser was irradiated at the center of the anodizing specimen for one h, red-PL appeared as strips distributed along the perimeter bands (Figure 4q) instead of on the irradiated spot. This effect may be attributed to the effect of free-carrier absorption^33^ in activating electrons, i.e., hot electrons, which increases the draining speed. After 3 h of anodization, the entire surface also exhibited the PL phenomenon. The PL peak locations on the anodized surface were almost the same at 600 nm at the irradiated spot, in the region near the irradiated spot, and in the unirradiated region. This result indicated that 1550 nm laser irradiation did not increase the degree of etching but accelerated the anodization of the entire surface, attributed to the free-carrier effect.^33^ The surface status of the 3 h laser-irradiated spot region was the same as that of the unirradiated region. After 3 h of anodization, the entire surface—including the nonirradiated area—exhibited PL, confirming that the Schottky junction effect induces hole self-doping.

**Figure 4:**
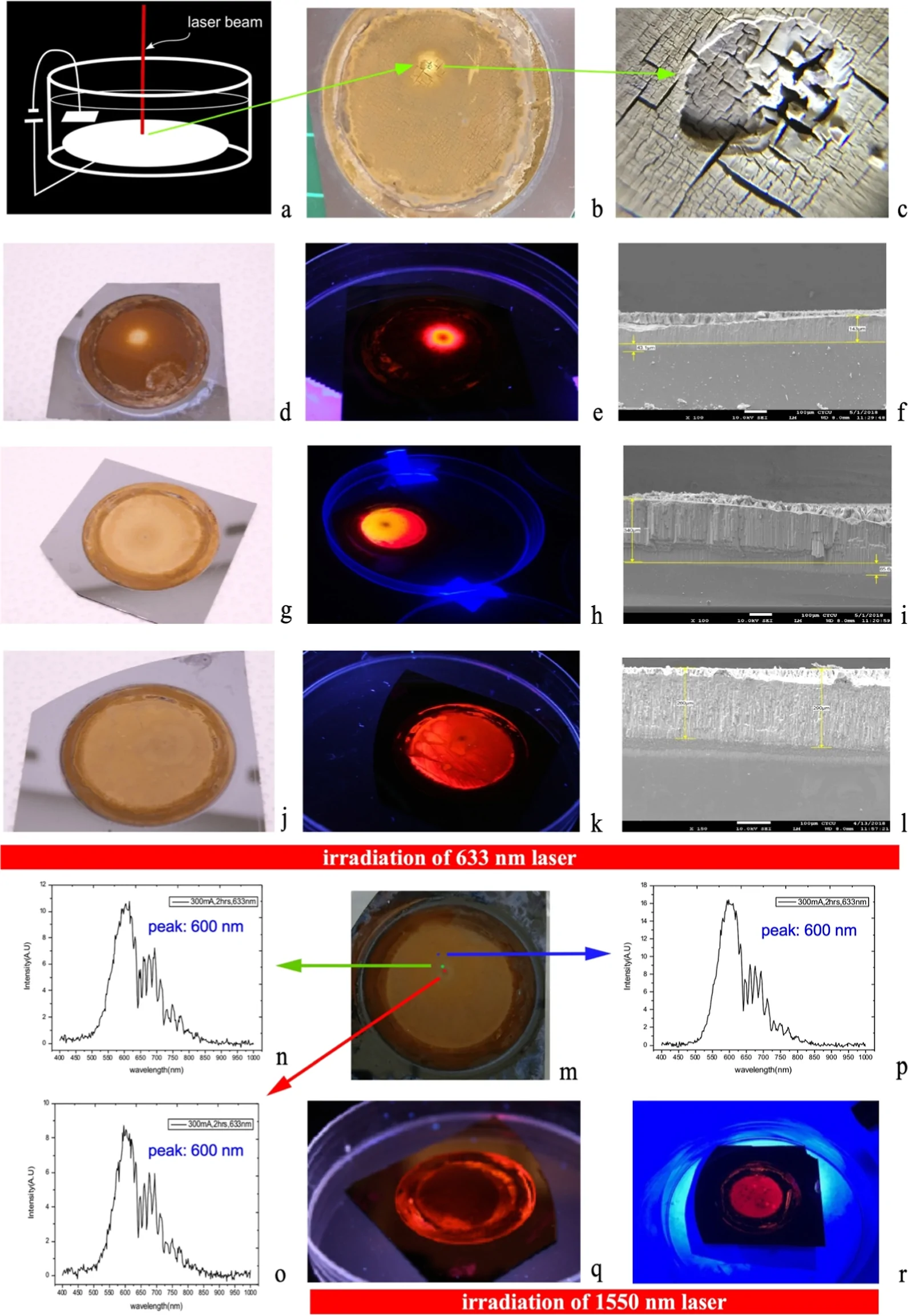
Experimental results of -type Si anodization under localized laser irradiation. (a) Schematic of the laser point-irradiation setup. (b–l) Surface morphology and cross-sectional views for 633 nm laser irradiation at 1, 2, and 3 h. (m–r) PL images and corresponding spectra for 1550 nm laser irradiation, comparing the irradiated spot, near-spot, and unirradiated regions.

While the entire surface of *n*-type silicon can be anodized in the dark at an appropriate current density, the process is intrinsically slow, typically requiring more than 1 h to initiate. We propose a mechanism attributed to the Schottky junction effect at the Si/metal interface, which facilitates a “hole-self-doping” process across the substrate to enable anodization. As shown in Figure 5, a comparative mechanism study using laser irradiation at 633 and 1550 nm of wavelengths reveals that localized irradiation significantly reduces the initiation time for the entire surface, including dark regions, compared to pure dark conditions. The effect of all laser types confirms the Schottky junction effect and that a localized laser trigger induces a surface-wide electrochemical reaction via the free carrier effect of holes. The following mechanical details illustrate the different manifestations of the Schottky-driven effect compared to general cognition: (1) the interplay between the Schottky junction effect and external irradiation significantly alters the spatial kinetics of the anodization process. Under supra-bandgap laser irradiation (*E*_laser_ *> E*_*g*_; e.g., λ = 633 nm), the anodization reaction initiates at the localized irradiated spot due to photogenerated carriers and subsequently propagates across the entire substrate surface as the hole-self-doping effect takes hold. The high concentration of photogenerated holes results in severe localized etching within the irradiated region. (2) In contrast, under sub-bandgap irradiation (*E*_laser_ *< E*_*g*_; e.g., λ = 1550 nm) or in complete darkness, the reaction follows a different nucleation pattern. In these cases, anodized regions initially emerge as disparate, isolated clusters scattered across the silicon surface. (3) Under complete darkness, these initial clusters are noticeably smaller in scale compared to those formed under sub-bandgap laser stimulation. With sustained anodic polarization, these independent domains gradually coalesce as the inversion layer expands, eventually forming a uniform, continuous porous layer across the entire surface.

**Figure 5:**
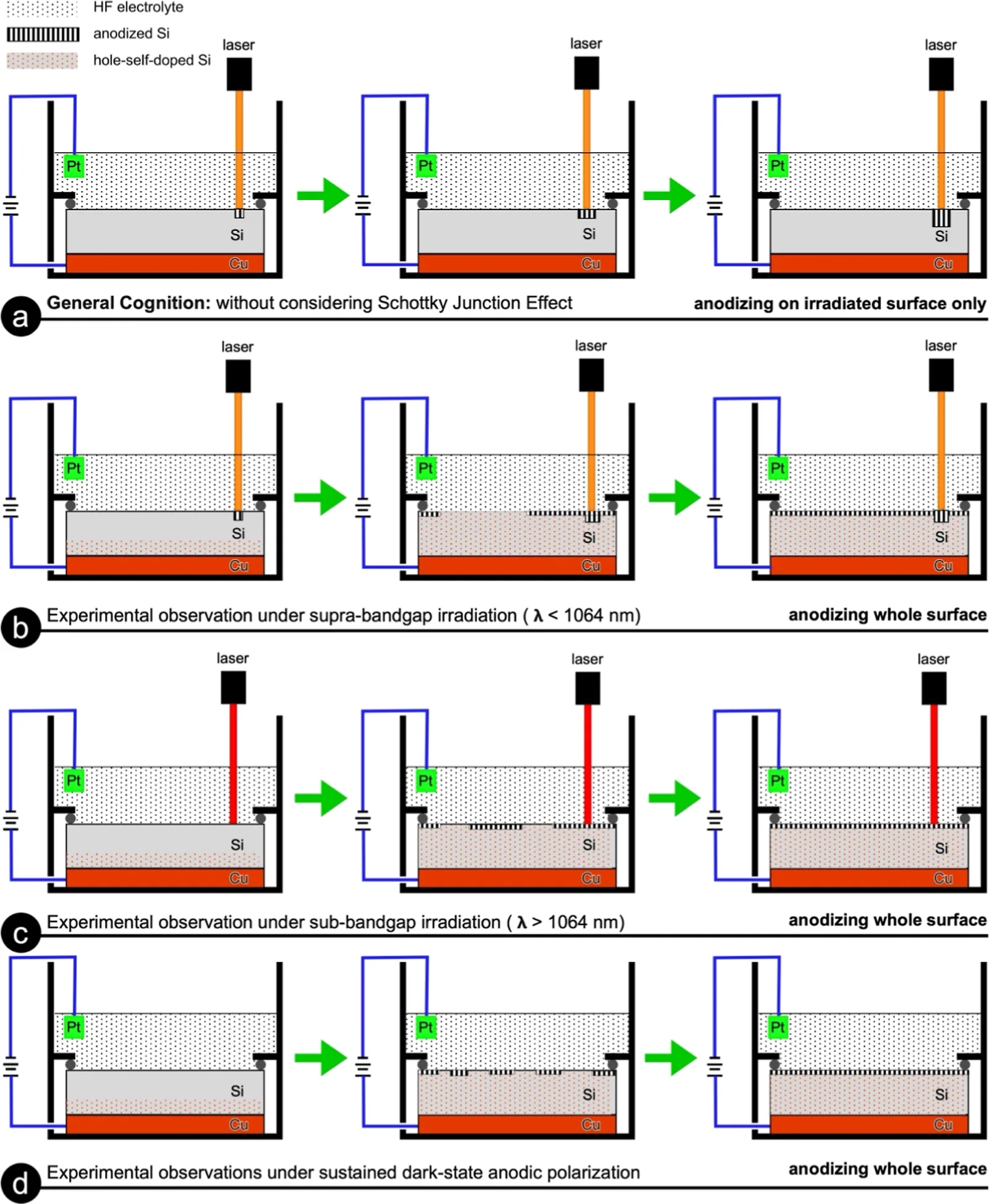
Schematic illustration of the Schottky-driven mechanism for*n*-type silicon anodization. (a) Conventional model. (b–d) Evolution of surface-wide anodization under supra-bandgap irradiation, sub-bandgap irradiation, and sustained dark-state anodic polarization, illustrating the transition from isolated clusters to a continuous anodized layer.

### Mechanism of Hole Self-Doping via Schottky-Driven Electron Extraction at the Metal Electrode/*n*-Type Semiconductor Interface

Figure 6 illustrates the proposed mechanism in which Schottky effect-induced hole self-doping enables the fluorination anodization of *n*-type silicon in the dark. Through a direct wafer-bonding process that joins Si and Cu electrodes, a Schottky junction develops at the well-bonded Cu/Si interface. At equilibrium, band bending at this junction creates a barrier that suppresses electron transfer from silicon to copper. After the Cu anode is connected to the positive terminal—effectively applying a forward bias—the reshaped band bending facilitates the extraction of electrons under an electric field, which act as majority carriers that flow into the copper at the interface. A sustained forward bias continuously removes electrons from the conduction band of *n*-type silicon, creating an electron-depleted region. This depletion reduces the local Fermi level and promotes net hole accumulation near the quasi-Fermi level, effectively inversing into a quasi-*p*-type region in the Si adjacent to the Cu electrode. The resulting quasi-*pn* junction (quasi *p-type* region/*n-*type Si bulk) facilitates net hole transport toward the Si/HF electrolyte interface under bias. The electrical double layer may attract F^–^ ions and let them physisorb onto the silicon interface. When enough holes accumulate at the electrolyte/silicon interface, fluorination occurs, resulting in the formation of volatile SiF_4_ (Lehmann–Gösele model). The longer delay in PL onset observed during *n*-type Si anodization than during *p-*type Si anodization suggests that the process is controlled by the rate of net holes—specifically, the extraction of majority carriers (electrons) into the Cu electrode via the Schottky junction. At a current of 100 mA (corresponding to a current density of 86 mA/cm^2^ for a 1.16 cm^2^ wafer), both lightly and heavily doped n-type silicon required over 3 hs of anodization to exhibit visible PL. Increasing the current to 300 mA and 500 mA (yielding current densities of 256 mA/cm^2^ and 428 mA/cm^2^, respectively) reduced the PL onset time to approximately 2 hs and 1 h. We conclude that higher current densities proportionally shorten the time required to reach PL onset and deepen the porous structures observed in FE-SEM cross-sectional images, as shown in Figure A3 of the Supplying Information. This proportional trend supports the hypothesis that PL appearance—and hence quantum-confined nanocrystal formation—is governed by the time required to deplete electrons from the silicon surface. On the basis of these results, we propose an “electric-field budget” for electrochemical reaction—analogous to the “thermal budget” in thermal activation—that captures the cumulative effect of applied bias and duration in enabling porous structure formation.

**Figure 6:**
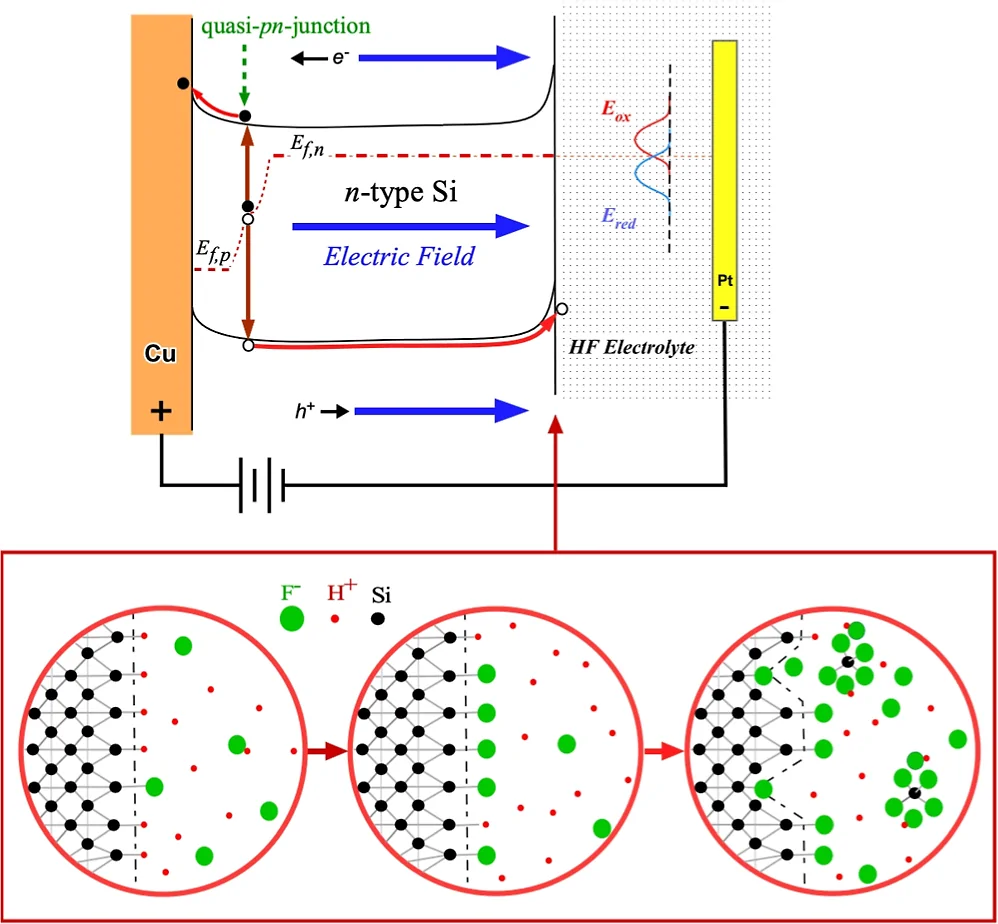
Schematic of hole self-doping and interfacial reactions.

With Cu as the anode, continuous electron depletion occurs in the *n-*type silicon under sustained bias. Under anodic polarization of the *n-*type Si/HF electrolyte interface, electron extraction and a decrease in the interfacial potential induce strong band bending, resulting in the formation of a space-charge (depletion) region in the silicon. With increasing surface potential, the near-surface region can enter surface inversion, where the interfacial hole concentration becomes sufficiently high to behave as a quasi-*p*-type inversion layer on *n*-type Si. Because silicon anodic oxidation/dissolution in HF is hole-driven, the onset and rate of anodization are controlled by hole availability and transport to the interface (drift–diffusion and generation–recombination), rather than atomic ionization energies of the dopants. These holes then drive surface silicon atoms to react with attached F^–^ ions, forming volatile SiF_4_ clusters that subsequently react with HF to yield soluble H_2_SiF_6_ in the electrolyte. This interfacial reaction is illustrated in the lower sequence of Figure 6.

Quantitative estimation indicates that the hole density after 3 h of processing exceeds the original carrier concentration by 2 orders of magnitude (for 1.0 Ω-cm, [*n*] ≈ 5 × 10^15^; for 0.005 Ω-cm, [*n*] ≈ 3.6 × 10^19^). This estimation confirms that the quasi-*p*-type transition induced by hole self-doping under a sustained bias governs both the onset and the rate of anodization in the dark. To quantitatively validate the Schottky junction-driven mechanism, the experimentally observed current densities (17 ∼ 86 mA/cm^2^) are reconciled with the theoretical hole flux *J* governed by carrier transport across the interfacial barrier. Under the high-bias conditions used in this study (20 V–40 V), the hole generation rate can be modeled by a modified Richardson–Dushman equation based on thermionic emission theory.

In this framework, the current density (*J*) under forward bias (*V*) is defined according to eqs 5 and 6

5 $$J=J_{0}\exp\left(\frac{qV}{nkT}\right)\left[1-\exp\left(\frac{qV}{kT}\right)\right]$$

and

6 $$J_{0}=AT^{2}\exp\left(-\frac{q\phi_{B0}}{kT}\right)$$

where *V* is the forward bias, *J*_0_ is the saturation current density, *q* is the electron charge, *k* is the Boltzmann constant, *T* is the absolute temperature, *A* is the effective Richardson constant of 110 A/(cm^2^ K^2^) for *n*-type Si, ϕ_*B*0_ is the zero bias apparent barrier height, and *n* is the ideality factor (=1 at 295 K). The saturation current density, derived by extrapolation of the linear forward bias region, was found to be 0.02 A/cm^2^ at 295 K. This correlation confirms that the electron-draining efficiency at the Cu/Si interface provides a sufficient hole flux to sustain the electrochemical kinetics of the Lehmann–Gösele pathway, even in complete darkness.

The mechanism of dark anodization originates from dynamic charge reconstruction at the metal–semiconductor interface, as illustrated in Figure 6. Under an applied bias, electrons are extracted from *n*-type Si across the Cu/Si Schottky junction into the copper anode, creating an electron-depleted region adjacent to the interface. As depicted in the band diagram in Figure 6, this majority-carrier extraction lowers the hole quasi-Fermi level (*E*_*f,p*)_ toward the valence band, thereby increasing the local hole density and inducing an inversion layer that subsequently evolves into a functional quasi*-p-*junction. This junction efficiently channels internally generated holes toward the Si/HF electrolyte boundary. The lower panels of Figure 6 depict the stepwise surface chemistry: fluoride ions (F^–^) are first physisorbed onto the Si surface, and once the hole concentration exceeds the activation threshold, they initiate fluorination. This reaction replaces hydrogen in surface dangling bonds and generates volatile SF_4_clusters, which desorb and subsequently react with the electrolyte to form solvated H_2_SiF_6_. The measured saturation current density (*J*_0_ ≈ 0.02 A/cm^2^) confirms that the electron-drain pathway provides a sufficiently strong hole flux to sustain the reaction kinetics even in complete darkness.

The top panel shows the evolution of the band diagram at the Cu/Si interface, highlighting a quasi-*p*-type transition as the Fermi level approaches the valence band edge, while the lower panels provide a stepwise visualization of the hole-mediated fluorination of surface silicon atoms leading to H_2_SiF_6_ formation.

This mechanism explains why *n-*type silicon, although initially inactive in darkness, begins to exhibit anodic dissolution after prolonged bias. The delay time before observable etching or photoluminescence correlates directly with the rate of electron extraction: higher current densities accelerate the depletion of electrons and shorten the onset time for pore formation. Once the inversion layer extends across the entire interfacial region, anodization proceeds rapidly and uniformly, producing nanostructured silicon that displays strong quantum-confined photoluminescence even in the absence of illumination.

The Schottky-junction-driven quasi-*p*-type transition therefore provides an intrinsic hole source for dark anodization. This transition operates analogously to conventional forward-biased *p*-type Si anodization but essentially differs in its origin: instead of injecting holes from an external circuit or via photoexcitation, holes are generated internally by the gradual electron drainage through the metal/semiconductor Schottky barrier. This process can be viewed as a form of self-doping, where the sustained electric field effectively redefines the local carrier population. The concept introduces an “electric-field budget” for electrochemical processing, analogous to the thermal budget in semiconductor manufacturing, in which the cumulative product of bias and time determines the extent of carrier inversion and, consequently, the etching kinetics.

From a broader perspective, this mechanism unifies the anodization behaviors of *p*-type and *n-*type semiconductors within a single electrostatic framework. In both cases, the anodic dissolution at the Si/HF interface is governed by the availability of hole carriers capable of breaking Si–H bonds and enabling fluoride complexation. With respect to *p*-type Si, holes are majority carriers that are readily supplied under forward bias. With respect to *n*-type Si, the same reaction pathway emerges only after a quasi-*p-*type inversion region develops because of sustained electron extraction at the Schottky interface. This unified interpretation reconciles the long-standing paradox that *n*-type silicon cannot be anodized in the dark and establishes a consistent physical picture in which charge redistribution—not illumination—is the decisive factor enabling hole-mediated fluorination anodization.

## Conclusions

Our results establish that the metal–semiconductor interfacial band structure can serve as an intrinsic hole source by regulating electron extraction, injection, and accumulation, thereby enabling a light-independent electrochemical platform for silicon nanostructuring.

Through probed laser-irradiation experiments, we show that the Schottky junction—rather than photogenerated carriers—is the primary driver of this surface-wide synthetic transformation. The junction induces “hole-self-doping,” enabling the fluorination anodization of *n*-type semiconductors without external hole injection or illumination. Under sustained anodic bias, continuous electron extraction at the Si/Cu interface generates an electron-deficient inversion layer at the metal–semiconductor boundary. This process results in net hole accumulation at the substrate–electrolyte interface, thereby sustaining the anodization reaction in complete darkness. Irradiation of small laser beams across three distinct wavelengths (633, 1064, and 1550 nm) yielded anodization and photoluminescence behaviors consistent with dark-anodization results, independently confirming that the Schottky junction effect controls internal charge redistribution. The successful anodization of both standard and heavily doped n-type silicon validates this mechanism, whereas the use of prime-grade wafers excludes contributions from surface traps or doping inhomogeneities. We quantify the efficacy of this approach through an “electric-field budget” framework—analogous to the “thermal budget” in semiconductor manufacturing—establishing that the cumulative product of bias and duration dictates pore formation and etching kinetics. The resulting silicon nanocrystals (3.2–3.4 nm in diameter) exhibit exceptional synthetic quality, with red–orange photoluminescence remaining stable for more than years under ambient conditions. This work uses a predictable, light-independent route for semiconductor nanostructuring, improving the nanofabrication toolkit for next-generation silicon photonics.

## Supplementary Material



## Notes

Schematic of the electrochemical setup, current–voltage (I–V) curves during processing, PL and FE-SEM characterization of dark anodization results, and comparative anodization results under irradiation of laser beams with wavelengths of 633, 830, and 1064 nm (PDF)
