# Supplementary material for: Fluorination Anodization of n‑Type Silicon in the Dark via Schottky-Driven Electron Extraction
Source: ACS Mater Au. 2026 Mar 27;6(5):1077–86. doi: 10.1021/acsmaterialsau.6c00002 (PMC13564145; doi:10.1021/acsmaterialsau.6c00002)
Supplement: Supplementary file 1 [file mg6c00002_si_001.pdf]

Supplying Information

Fluorination Anodization of n-Type Silicon in the Dark via Schottky-Driven Electron Extraction

Benjamin Tien-Hsi Lee[1] *, Chao-Chia Cheng[2], Chun-Huang Wu[1], Lin Cheng[1], Yu-Sheng Chiou[1], Shu-Cheng Li[1], Wei-Chi Huang[1], Chao-Yuan Cheng[1], Yang-Chun Chiu[3], and Chao-Ching Chiang[4]

[1] Department of Mechanical Engineering, National Central University, Taoyuan City 320317, Taiwan Republic of China

[2] Department of Physics, National Central University, Taoyuan City 320317, Taiwan, Republic of China

[3] Department of Materials and Mineral Resources Engineering, National Taipei University of Technology, Taipei 10608, Taiwan, Republic of China

[4] Department of Mechanical Engineering, Asia Eastern University of Science and Technology, New Taipei 220303, Taiwan, Republic of China

Experimental procedure

(1) n-Type silicon was cut and cleaned with RCA1 (NH_4OH:H_2O_2:H_2O = 1:1:5) and RCA2 (HCl:H_2O_2:H_2O = 1:1:5) at 85°C for 10 minutes.
(2) The cleaned Si specimens were anodized using an electrolyte (HF:C_2H_5OH = 1:1) under dark conditions.
(3) The specimens were observed by PL imaging and measurement.
(4) The specimens were observed by TEM and FE-SEM.

Photoluminescence measurements

(1) Instrument: Horiba T64000 micro-PL/Raman spectrometer
(2) He-Cd laser, wavelength: 325 nm, power: 3.5 mW

UV lamp

Manufacturer: Cool-Touch™ lamp heads UVP
Wavelength: 365 nm
Power: 100 W

Calculation of concentration of hole input into silicon during anodization

Assumption: The amount of lateral current was negligible because the cross-sectional area was very small.
Conditions: (1) Sheet resistivity = 1.0 ohm-cm ≈ $[n] = 5 \times 10^{15}$; 0.005 ohm-cm ≈ $[n] = 3.6 \times 10^{19}$, (2) current = 0.5 A, (3) anodization time = 3 hours, (4) anodization area = 9.8 cm^2, (5) wafer thickness = 400 μm = 0.04 cm

The total number of input holes $= 0.5 \times 3 \times 60 \times 60 \div (1.6 \times 10^{-19}) = 3.375 \times 10^{22}$
The concentration of input holes (number/cm^3)
$= 3.375 \times 10^{22} \div (9.8 \times 0.04) = 8.6 \times 10^{22}$ ($> 3.6 \times 10^{19}$ and 5×10^{15})

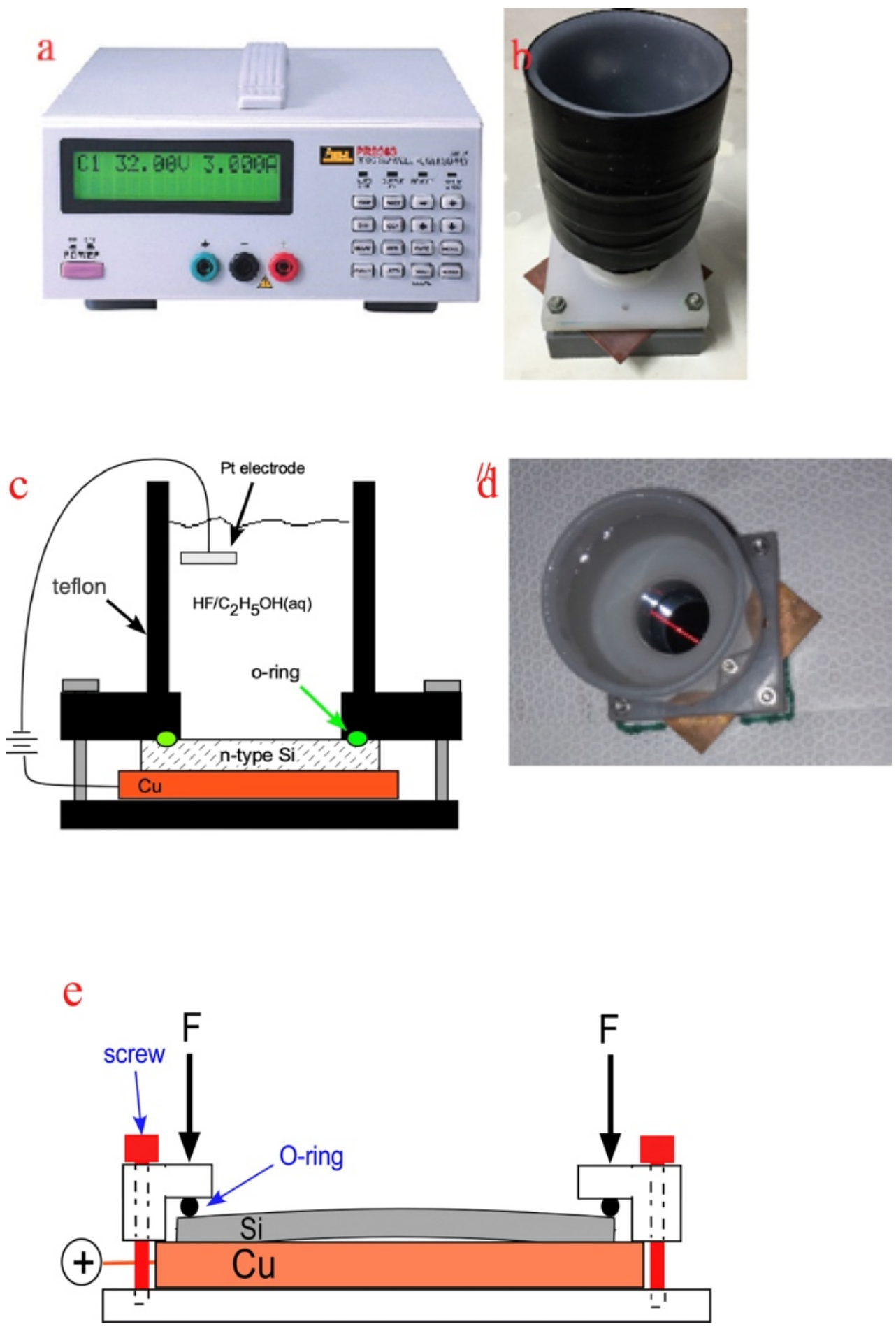

Figure A1: The equipment setup includes a power supply (a) and a Teflon etching tank wrapped in black tape to keep light out (side view (b) and top view (d)). The structural diagram of the Teflon etching tank shows the O-ring used to effectively prevent electrolyte leakage into the interface of the copper electrode and the silicon substrate to be anodized. When the four outer screws next to the O-ring are tightly secured, the center of the silicon substrate can be slightly arched and lose contact with the copper (e).

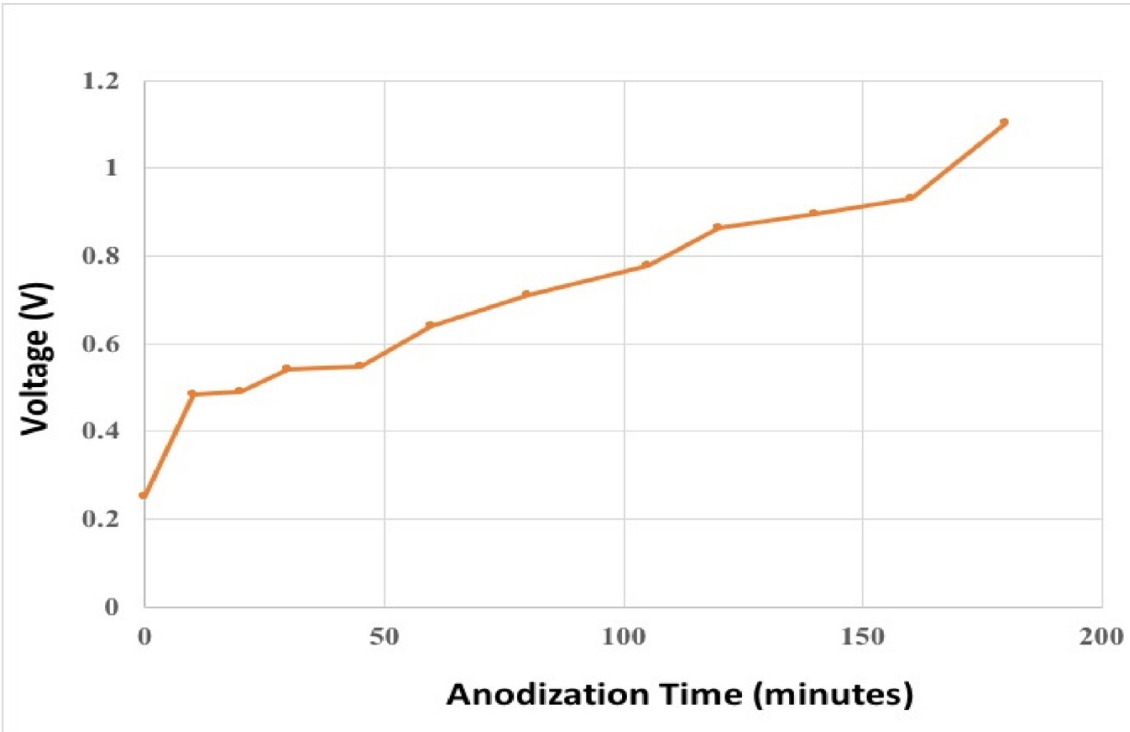

a

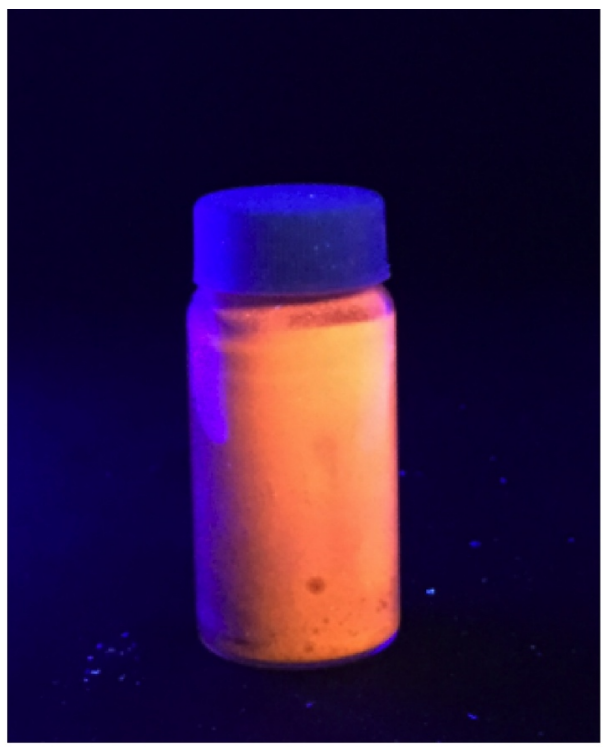

b

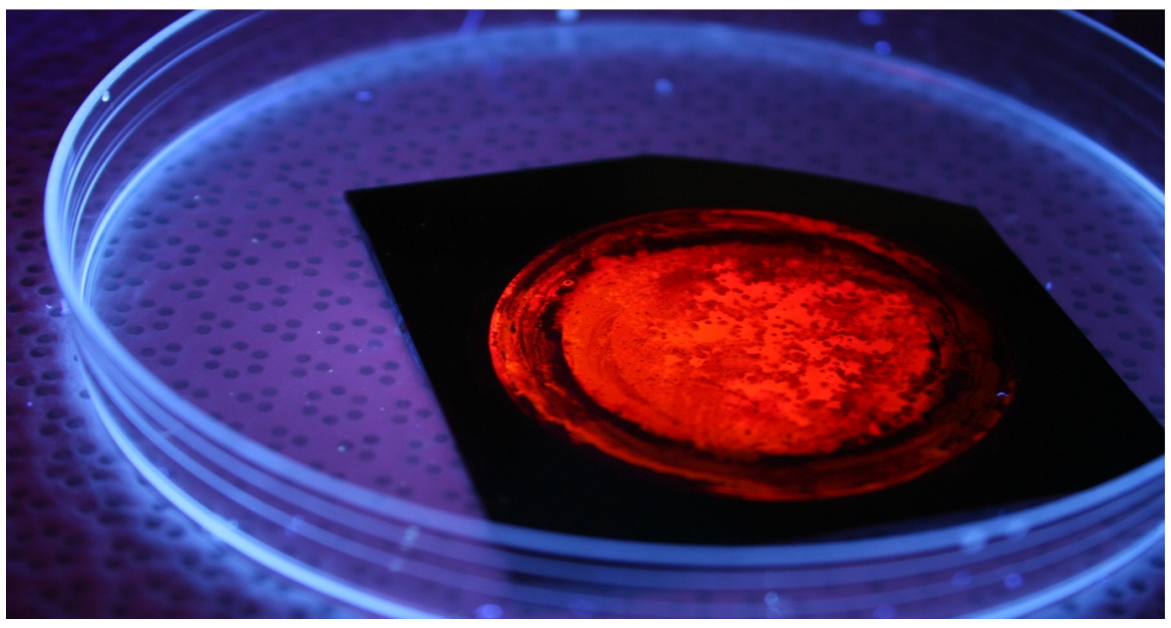

c

Current Density (mA/cm^2)	Time (hour)
86	3
120	2
256	0.5

d

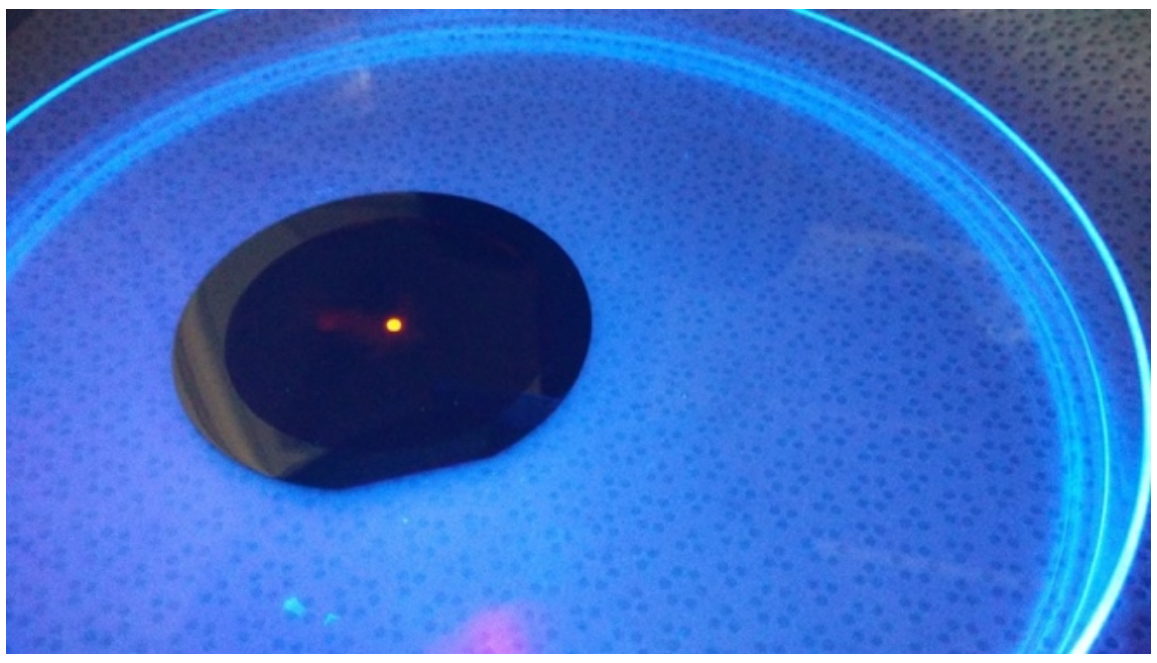

(e)

Figure A2: Chart, images and table of n^{++}-type Si anodization in the dark. Under a constant voltage, the applied current varied with processing time (a). Nanocrystals of anodized Si collected in a glass bottle (b). A current density of 256 mA/cm^2 was applied for 30 minutes to anodize regular n-type Si and attain the PL phenomenon under UV irradiation (c). Table of the relationship between current density and time for achieving PL results after the anodization of n-type silicon in the dark (d). Anodization of a p^{++} silicon substrate with a 633 nm laser spot irradiation; a bright yellow-spot formed on the black anodized surface under irradiation with UV light (e).

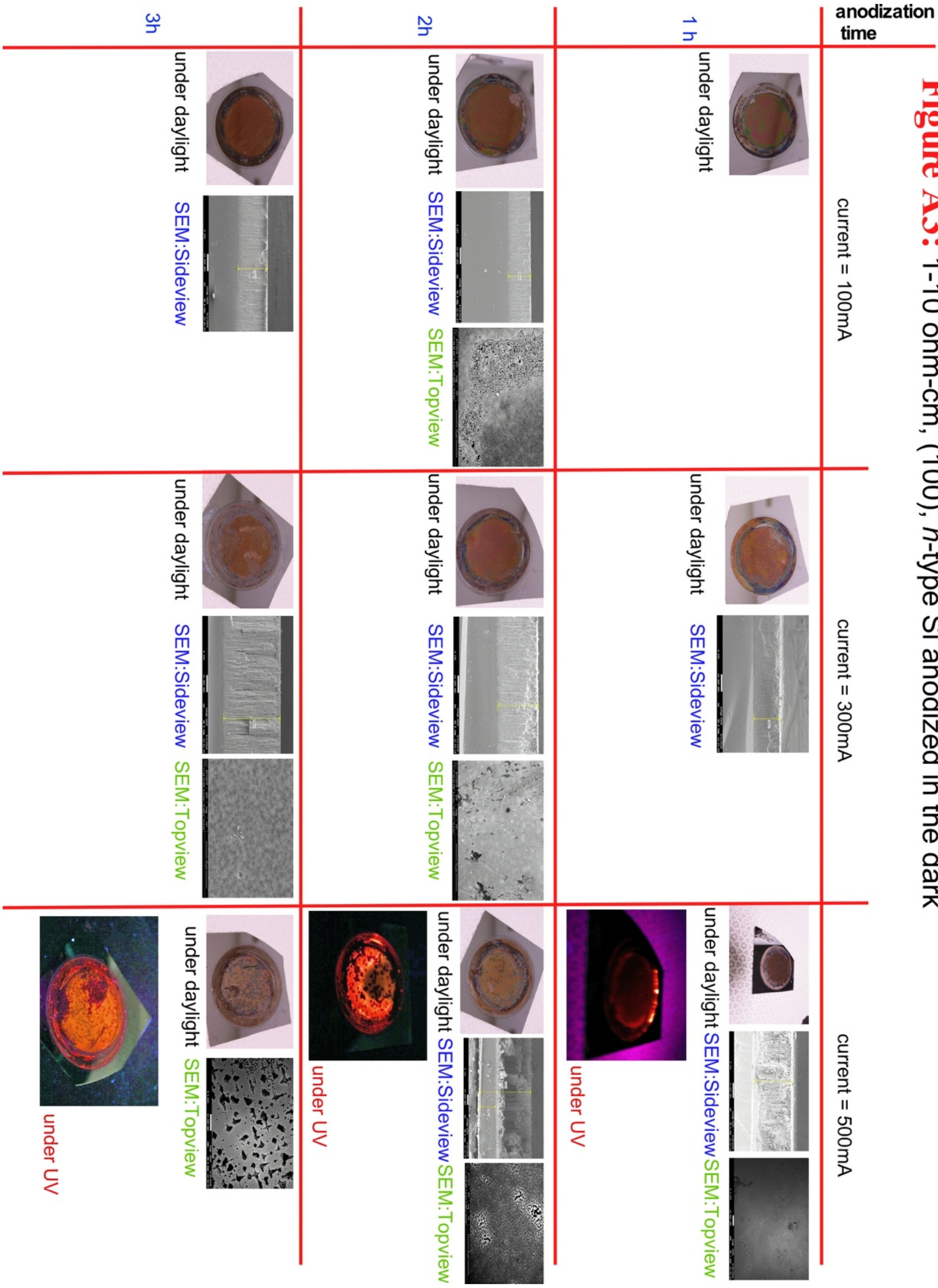

Figure A3: PL and FE-SEM images of *n*-type Si after anodization in the dark. The experimental results are sufficient to prove that the anodization of *n*-type Si can occur in the dark due to the Schottky junction.

The experimental results demonstrate anodization (e.g., increased oxide thickness or changes in surface morphology) under dark conditions, it would support

the idea that the Schottky junction plays a crucial role. The presence of measurable electrochemical reactions, such as current flow or potential shifts, would further substantiate this claim.

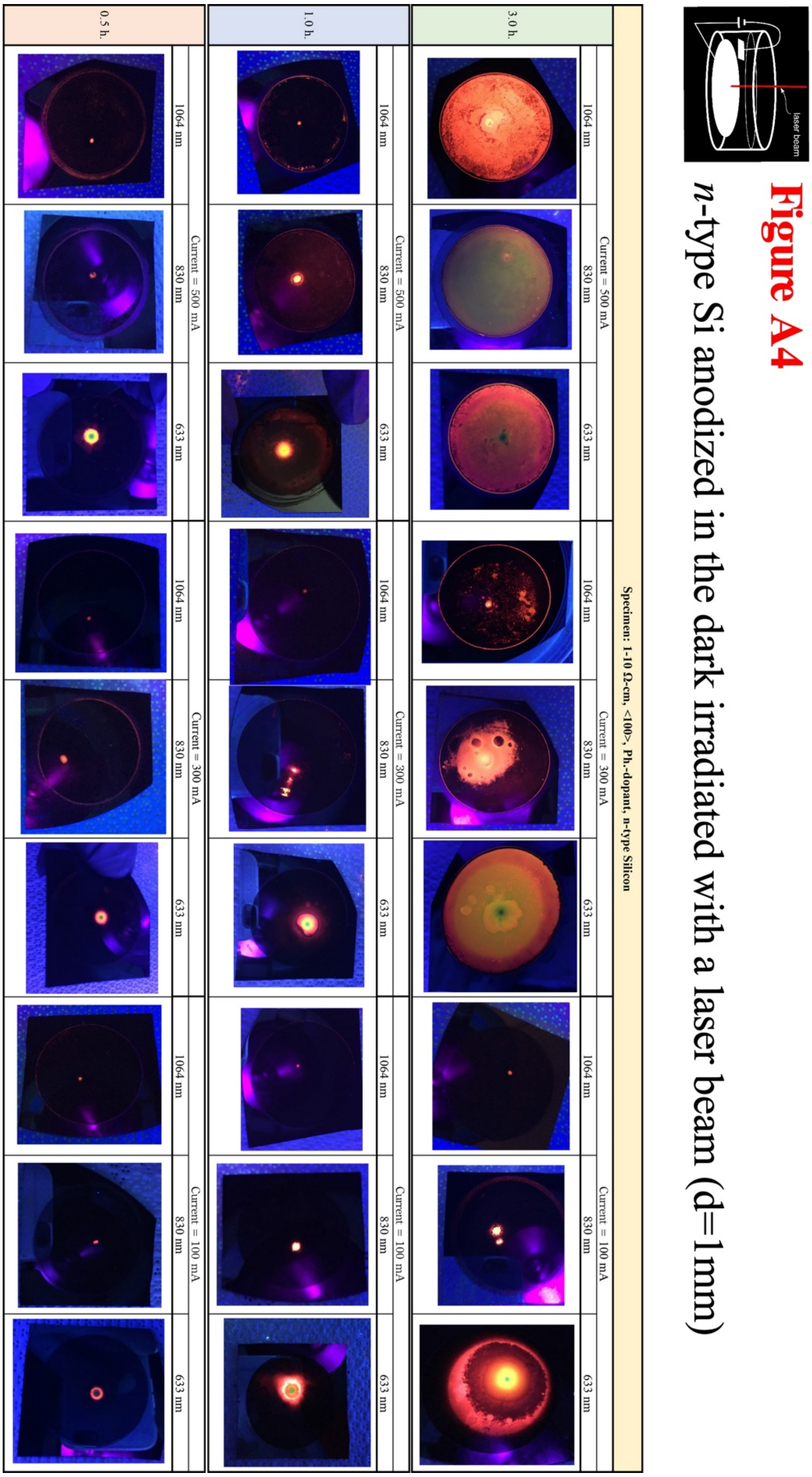

Figure A4: The 633 nm laser can represent this proposed mechanism as an example because the n-to-p transformation region (whole anodized area, red PL) is much larger

than the region exhibiting interband absorption (the irradiated point, yellow PL). At 1064 nm, the interband absorption effect is still observed at the critical wavelength; however, it disappears and is not different from the unirradiated area when irradiated at 1550 nm. The experimental results of irradiation with lasers with wavelengths of 633, 830, 1064, and 1310 nm demonstrate that the anodization of the silicon surface is very sensitive to the concentration of electronic holes.
